# Load-sharing characteristics of stenting and post-dilation in heavily calcified coronary artery

**DOI:** 10.1038/s41598-023-43160-4

**Published:** 2023-10-06

**Authors:** Pengfei Dong, Jose Colmenarez, Juhwan Lee, Neda Shafiabadi Hassani, David L. Wilson, Hiram G. Bezerra, Linxia Gu

**Affiliations:** 1 Department of Biomedical Engineering and Science, Florida Institute of Technology, Melbourne, FL 32901 USA; 2 Department of Biomedical Engineering, Case Western Reserve University, Cleveland, OH 44106 USA; 3 Cardiovascular Imaging Core Laboratory, Harrington Heart & Vascular Institute, University Hospitals Cleveland Medical Center, Cleveland, OH 44106 USA; 4 Interventional Cardiology Center, Heart and Vascular Institute, The University of South Florida, Tampa, FL 33606 USA

**Keywords:** Medical research, Biomedical engineering

## Abstract

In this work, stenting in non-calcified and heavily calcified coronary arteries was quantified in terms of diameter-pressure relationships and load transfer from the balloon to the artery. The efficacy of post-dilation in non-calcified and heavily calcified coronary arteries was also characterized in terms of load sharing and the changes in tissue mechanics. Our results have shown that stent expansion exhibits a cylindrical shape in non-calcified lesions, while it exhibits a dog bone shape in heavily calcified lesions. Load-sharing analysis has shown that only a small portion of the pressure load (1.4 N, 0.8% of total pressure load) was transferred to the non-calcified lesion, while a large amount of the pressure load (19 N, 12%) was transferred to the heavily calcified lesion. In addition, the increasing inflation pressure (from 10 to 20 atm) can effectively increase the minimal lumen diameter (from 1.48 to 2.82 mm) of the heavily calcified lesion, the stress (from 1.5 to 8.4 MPa) and the strain energy in the calcification (1.77 mJ to 26.5 mJ), which are associated with the potential of calcification fracture. Results indicated that increasing inflation pressure can be an effective way to improve the stent expansion if a dog bone shape of the stenting profile is observed. Considering the risk of a balloon burst, our results support the design and application of the high-pressure balloon for post-dilation. This work also sheds some light on the stent design and choice of stent materials for improving the stent expansion at the dog bone region and mitigating stresses on arterial tissues.

## Introduction

Stenting in heavily calcified coronary arteries is challenging due to the inherent residual stent underexpansion and malapposition. Post-dilation with a shorter balloon at a higher inflation pressure has been widely adopted to improve the stent expansion. However, it increases the risk of intima dissection and vessel rupture^1,2^. A retrospective study has scored stent sizing, balloon post-dilation, and pre-dilation to predict one-year adverse cardiac events following implantations of bioresorbable stent^3^. So far, a similar scoring system is lacking for the metallic stents and the role of the pre- and post-dilation of the metallic stents is not well studied, even controversial. There is also mounting evidence to query if pre-dilation is necessary^4–6^. A clinical report of eight patients has demonstrated the efficacy of a non-compliant balloon with an ultra-high inflation pressure of 40 atm^7^. However, the contribution of high inflation pressure to arterial mechanics was not clear. In addition, no existing publications quantified how the inflation pressure is transferred from the balloon to the stent-lesion system. The physical characterization of stenting and post-dilation could help to address the aforementioned controversies and shed light on better pre-clinical planning.

The finite element (FE) method has been widely used to evaluate stent behaviors^8–10^ and predict vessel damage and adaptations^11–16^. Specifically, three-dimensional patient-specific artery models have been developed by coregistrating both optical coherence tomography (OCT) and computerized tomography (CT) images for performing structural and hemodynamic analyses^17^. It has been shown that the pre-dilatation and lesion compositions, such as lipid pool, fibrous cap, intraplaque hemorrhage, and calcification, affect the arterial responses^18–20^. The abnormally high arterial stresses, induced by the stent implantation, is positively correlated with the risk of restenosis^21,22^. Using patient-specific artery models, our group has systematically inspected the influence of the calcification attributes on the stenting outcomes, including stent underexpansion and malapposition^23,24^. Our FE results have illustrated that a larger calcification angle constricted the stretchability of the lesion, and thus the stent expansion capacity. An in-vitro uniaxial tensile experiment also demonstrated that a larger calcification volume leads to a reduced stretchability of the lesion^25^. We conducted additional ex-vivo and in-silico experiments to further test this indicator in the post-dilation procedures^26^. These observations directed our attention to the stretchability of the lesion, i.e., the amount of fibrous plaque along the circumferential direction, which might be the major determinant of stent expansion. With these accumulated studies on the correlation between lesion features and stenting outcomes, now it’s necessary to link the stenting outcomes in varied lesions with the required pressure for optimal stenting. So far as the authors know, the nominal pressure for a wire balloon is around 12 atm, cutting balloon 6–8 atm^27^, and drug-eluting balloon 7–8 atm^28^. Moreover, the BIB balloon has a burst pressure of only 7 atm or less^29^. The nominal pressure of a balloon to expand a stent, or a non-compliant (NC) balloon for post-dilation is usually 12 atm. We can see that the nominal pressure or rated burst pressure is varied for different balloon types, but the required load for expanding the same specific lesion should be the same with different balloon types. This indicates that not all pressure load is transferred to the lesion, quite an amount is undertaken by the balloon itself. So far, there are no quantitative studies on load transfer/sharing, which is essential for the balloon design and optimal stenting. Therefore, the quantification of load transfer characteristics during stenting and post-dilation procedures are in immediate needs for enhanced understandings of the stent-artery interaction and designing better clinical strategies.

The objective of this work is to quantify the load sharing among the balloon, stent, artery and lesion components (i.e., fibrous plaque, and calcification, if any), during stenting and post-dilation in non-calcified and heavily calcified coronary arteries. The pressure-diameter relationship of the balloon, and the radial force transferred to the balloon, stent and lesion will be characterized. Further, the efficacy of high-pressure post-dilation balloon will be inspected in terms of strain energy in each component and the maximum principal stresses in the artery. The fundamental understanding of load transfer and load sharing could better guide the optimal stenting in complex lesions.

## Materials and methods

### Model construction

**Figure 1:**
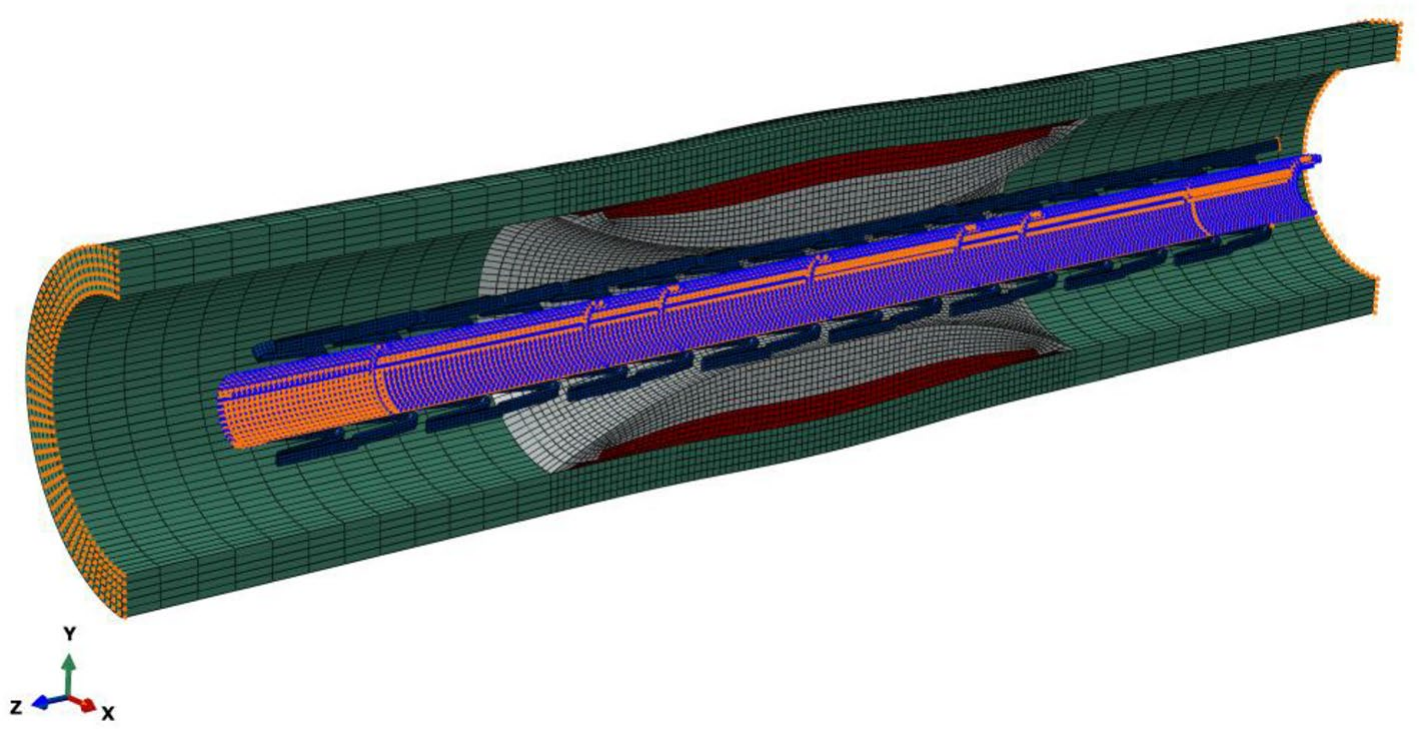
Geometric model of the heavily calcified coronary artery with a crimped Express stent. The fibrous plaque existed between calcification and artery. Symmetric boundary conditions in the Z-axis were applied to both ends of the artery, while only radial displacements were permitted in the balloon.

An idealized coronary artery was modeled as a cylinder with a length of 20 mm, an inner diameter of 3 mm, and a thickness of 0.5 mm^30^. The center-located plaque had a parabolic shape with a length of 8.5 mm and a minimum diameter of 1.2 mm, mimicking a diameter stenosis of 60% (i.e., area stenosis of 84%). The fibrous plaque was adopted for the case of the non-calcified lesion. For the case of the heavily calcified lesion, a block of calcification over a thin fibrous plaque was adopted, as shown in Fig. 1. The superficial concentric calcification had a maximum thickness of 0.64 mm, and the fibrous plaque had a maximum thickness of 0.26 mm. A commercial metal-based stent from the Express product line (Boston Scientific, Natick, MA, USA) was used. It has a length of 16 mm, a thickness of 0.13 mm and a nominal diameter of 3 mm at the nominal pressure of 12 atm. The NC balloon used to expand the stent was simulated as a cylinder with an initial diameter of 0.8 mm, a length of 17 mm and a diameter of 3 mm.

### Material properties

The hyperelastic behaviors of the artery, fibrous plaque, and calcification were described by the reduced third-order polynomial strain energy density function U:

1 $$U=\sum_{i,j=1}^{3}{C}_{ij}{({I}_{1}-3)}^{i}{({I}_{2}-3)}^{j}$$

2 $${I}_{1}={\lambda }_{1}^{2}+{\lambda }_{2}^{2}+{\lambda }_{3}^{2}$$

3 $${I}_{2}={\lambda }_{1}^{-2}+{\lambda }_{2}^{-2}+{\lambda }_{3}^{-2}$$

**Figure 2:**
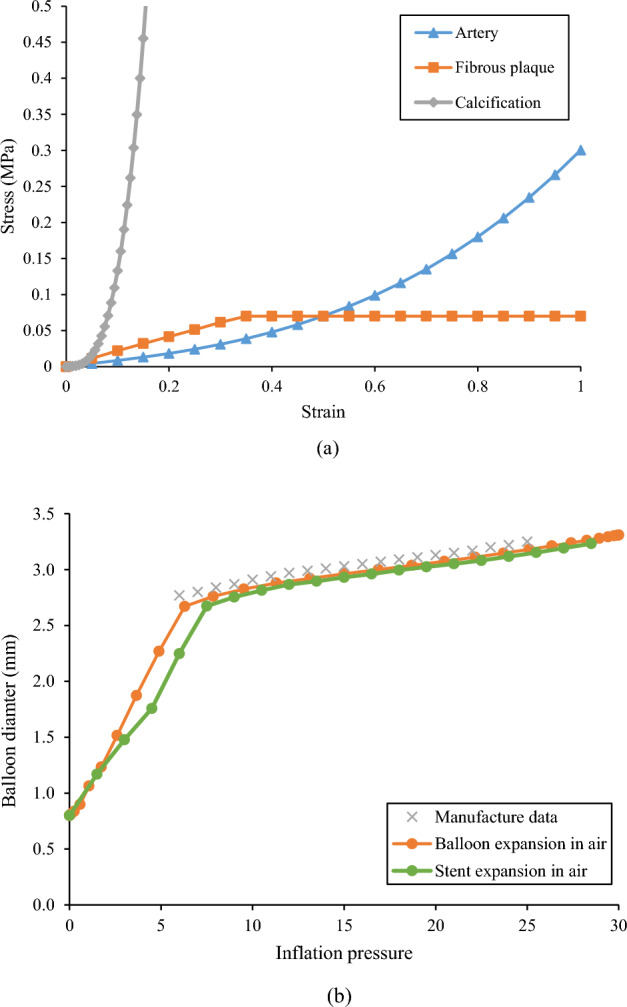
(**a**) Material properties of the artery, fibrous plaque, and calcification; (**b**) Pressure-diameter relationship between manufacturer data and simulation (balloon expansion, and stent expansion in the air).

The material coefficients C_ij_ were adopted from our previous work^31^, as shown in Table 1. A perfect plastic model was used to describe the tissue compaction of the fibrous plaque, which is necessary to capture a realistic stent expansion including stent recoil^32^. The plasticity of fibrous plaque was initiated at a strain of 34% when its stress reached its yield strength of 0.07 MPa^33^. The stress–strain relationship for all these lesion components is shown in Fig. 2a.

**Table 1:** Material coefficients.

	C_10_(MPa)	C_01_(MPa)	C_11_(MPa)	C_20_(MPa)	C_02_(MPa)	C_30_(MPa)	C_03_(MPa)
Artery	0.10881	–0.101	–0.1790674	0.0885618	0.062686		
Fibrous plaque	0.04				0.003		0.02976
Calcification	–0.49596	0.50661	1.19353	3.6378		4.73725

The pressure-diameter data of a Medtronic NC Euphora balloon was used to derive the material properties of the balloon (Fig. 2b). It is clear that the balloon exhibited a bilinear inflation behavior. The balloon diameter increased faster when the inflation pressure is below 6 atm, and much slower when the pressure exceeded 6 atm. To convert this bilinear inflation behavior of the balloon to a stress–strain relationship, the hoop strain was calculated as the relative change in diameter, and the hoop stress of a thin-walled cylinder was used to estimate the wall stress:

5 $$\upvarepsilon =\frac{{D-D}_{0}}{{D}_{0}}$$

6 $$\upsigma =\frac{PD}{2\delta }$$

where D is the diameter during expansion, D_0_ is the initial diameter of 0.8 mm, P is the inflation pressure, and δ is the thickness of the balloon. The pressure-diameter curve obtained from simulation was compared with the manufacturing data with maximum difference less than 5% for pressure interval from 6 to 25 atm (Fig. 2b).

The Express stent model incorporated elasto-plastic characteristics based on usual mechanical specifications of stainless steel 316L. These specifications encompass a Young’s modulus of 190 GPa, a Poisson’s ratio of 0.3, and a yield strength of 207 MPa. Beyond the yield point, a perfectly plastic response was assumed^34^.

Finite element simulations of the calcified coronary artery in the context of idealized or patient-specific models have been well validated in our previous work by matching the stented lumen area with ex-vivo experiments, or matching the simulated stress level with the published data^23,24,26^. As part of these studies, mesh convergence analyses were conducted, unveiling the consistency of the results when employing an element size smaller than 0.12 mm. Hence, a total 123,100 hexahedron elements were generated for the current study’s artery model in accordance with this criterion (convergence graph shown in the Supplementary Material). Symmetric boundary conditions (i.e., the displacement along the longitudinal direction is constrained, while along the transverse direction allowed) were applied to both ends of the artery such that the stenting procedure does not alter the lesion length far from the implantation site. In contrast, the balloon was constrained in all directions except for the radial component. For the stenting procedure, 10 atm was applied to the inner surface of the balloon. For the post-dilation, three pressures (i.e., 10 atm, 20 atm, and 30 atm) were sequentially applied to the inner surface of the balloon. General frictionless contacts were used for all interacting surfaces^35^. Energies were monitored during the stenting and post-dilation procedures to ensure that the dynamic effect (i.e., inertial forces) was acceptable. The ratio of the kinetic energy to the internal energy of the whole model was kept below 5%. The models were solved using Abaqus/Explicit 2022 (Dassault Systemes Simulia Corp., Providence, RI, USA), with each simulation taking approximately 7 h to complete.

Following simulation, the load transfer and load sharing were quantified. The load transfer refers to the action-reaction force between balloon, stent, and lesions. The load sharing refers to the strain energy stored in each component of the lesion and the stent. The pressure load is calculated as the radial force applied to the inner surface of the balloon:

7 $${F}_{r}=P\pi DL$$

where P is the inflation pressure, D is the diameter of the balloon, and L is the length of the balloon. The radial forces applied on the inner surface of the stent and artery were obtained by adding all the radial component of the contact force at each node on their inner surface.

## Results

Stenting in both non-calcified and heavily calcified lesions was compared. The load transfer from inflation pressure to the balloon, stent, and artery were quantified. The stress analysis and load sharing among the balloon, stent, and artery were used to further quantify the efficacy of the high-pressure balloon in improving the stent expansion in heavily calcified lesion.

### Stent expansion and pressure-diameter curve

**Figure 3:**
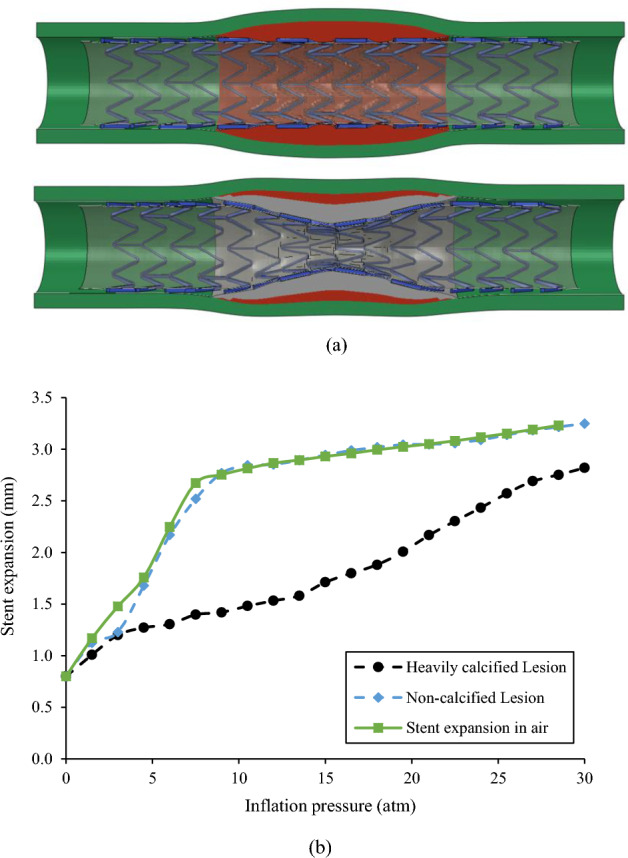
(**a**) Stent expansion profile in non-calcified (top) and heavily calcified (bottom) lesion; (**b**) Pressure-diameter relationship for stent expansion in the air, non-calcified lesion, and heavily calcified lesion.

The stent expansion in the non-calcified lesion and heavily calcified lesion are shown in Fig. 3. As inflation pressure increased to 10 atm, the stent expansion in the non-calcified lesion reached an inner diameter of 2.80 mm in the non-calcified artery, with a nearly cylindrical shape (Fig. 3a top). Stent underexpansion was observed in the heavily calcified artery, forming a dog bone shape with a minimal diameter of 1.48 mm (Fig. 3a bottom), leading to a residual stenosis of 49% in diameter and 74% in area. The associated pressure-diameter relationship is shown in Fig. 3b. The stent expansion in the non-calcified lesion was quite similar to the expansion in air, indicating the non-calcified lesion has minimal resistance and load sharing. On the contrary, the stent expansion in the heavily calcified lesion requires a much higher pressure beyond the normal pressure of 10 atm to obtain the predefined minimal diameter. As the inflation pressure increased from 10 to 30 atm, the minimal diameter of the non-calcified lesion increased from by only 19% (2.8 mm to 3.2 mm), while of the heavily calcified lesion it increased by 90% (from 1.48 to 2.82 mm).

### Radial force transferred from inflation pressure to balloon, stent, and lesion

**Figure 4:**
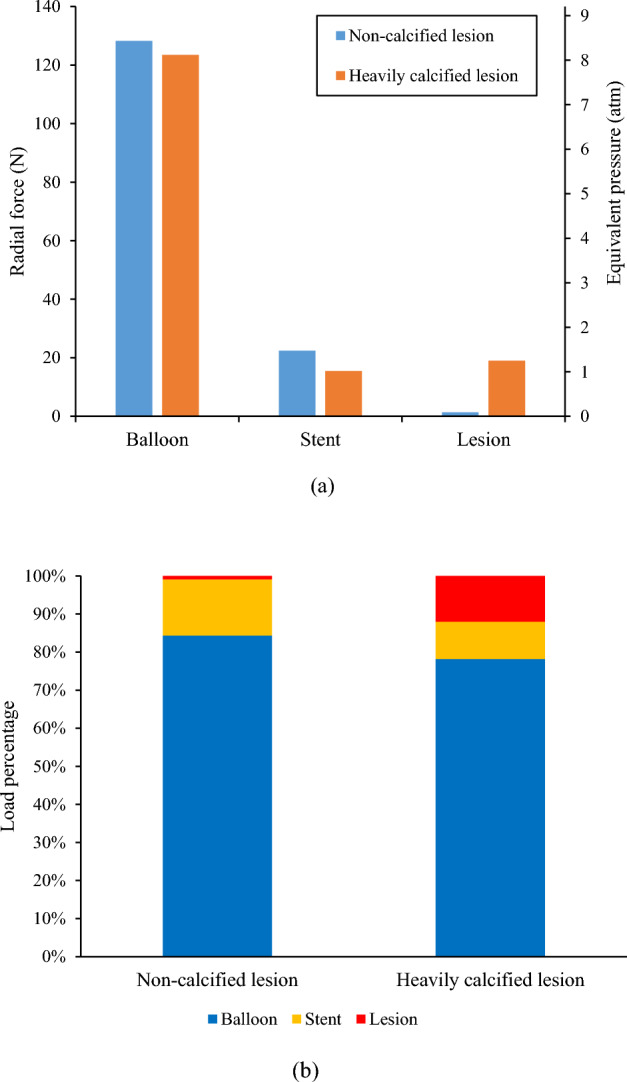
Radial force transferred from inflation pressure to the balloon, stent, and lesion in cases of stent expansion in non-calcified and heavily calcified lesions: (**a**) Clustered columns of radial force and equivalent pressure; (**b**) Stacked columns of pressure load percentage.

The radial force transferred from the inflation pressure to the balloon, stent, and lesion are depicted in Fig. 4. The inflation pressure of 10 atm (i.e., 1.013 MPa) resulted in a cylindrical expansion with an inner diameter of 2.76 mm (Fig. 3), corresponding to a pressure load of 152 N exerted onto the balloon surface. For stent expansion in the non-calcified lesion, a radial force of 23.8 N (i.e., 15% of the pressure load) was transferred to the stent and lesion, of which 1.4 N (i.e., 0.8% of the pressure load) was transferred to the non-calcified lesion. For stent expansion in the heavily calcified lesion, a radial force of 34.5 N (i.e., 22% of the pressure load) was transferred to the stent and lesion, and 19 N (i.e., 12% of the pressure load) was transferred to the lesion. We could convert the radial force transferred to the lesion to an equivalent pressure using Eq. 7. Therefore, a pressure of 0.092 atm (i.e.,70 mmHg) was transferred to the non-calcified lesion, while a pressure of 1.25 atm (i.e., 950 mmHg) was transferred to the heavily calcified lesion. It should be noted that all the above load calculation is based on cylindrical shape assumption. Considering the actual dog-bone shape in the case of the heavily calcified lesion, the pressure load was less than 152 N, but the difference was less than 10%.

### Load sharing in terms of strain energy during stenting and post-dilations

**Figure 5:**
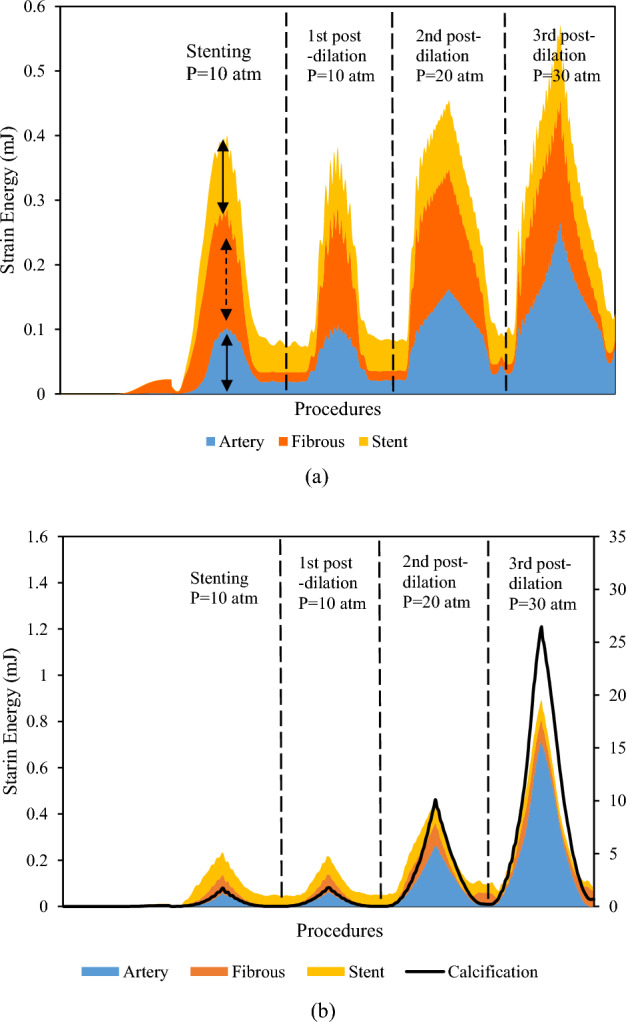
Stacked area graph of strain energy following stenting and post-dilations in (**a**) non-calcified lesion and (**b**) heavily calcified lesion. The right axis is for calcification curve only.

Strain energy stored in the artery, fibrous plaque, calcification (if any), and stent during the stenting and post-dilation procedures are shown in Fig. 5. The stacked areas are used to show how each component contributes to the total strain energy of the stented lesion. It is clear that, regardless of the lesion type, the absorbed strain energy in all components reached its peak at each target inflation pressure, and then recoiled back following the balloon deflation. For the non-calcified lesion, the peak strain energy of fibrous plaque was 0.184 mJ at the full expansion of the stent (arrow in Fig. 5a), and was 0.182 mJ, 0.184 mJ, 0.189 mJ, respectively, during three sequential post-dilations with increased inflation pressures. The peak strain energy stored in both stent and lesion was 0.35 mJ during stenting and increased to 0.55 mJ during the 3^rd^ post-dilation at the inflation pressure of 30 atm. In the heavily calcified lesion, the corresponding total strain energy stored in the stent, fibrous plaque and artery was 0.21 mJ during stenting and sharply increased to 0.90 mJ during the 3^rd^ post-dilation at inflation pressure of 30 atm. The peak strain energy in the calcification alone (labeled in the right y-axis of Fig. 5b) was 1.77 mJ, and increased to 26.5 mJ (15 times) during the 3^rd^ post-dilation at the inflation pressure of 30 atm, making it substantially higher than the other components. The huge increase of the strain energy in calcification indicates a potential fracture.

### Maximum principal stresses induced in lesions after stenting and post-dilations

**Figure 6:**
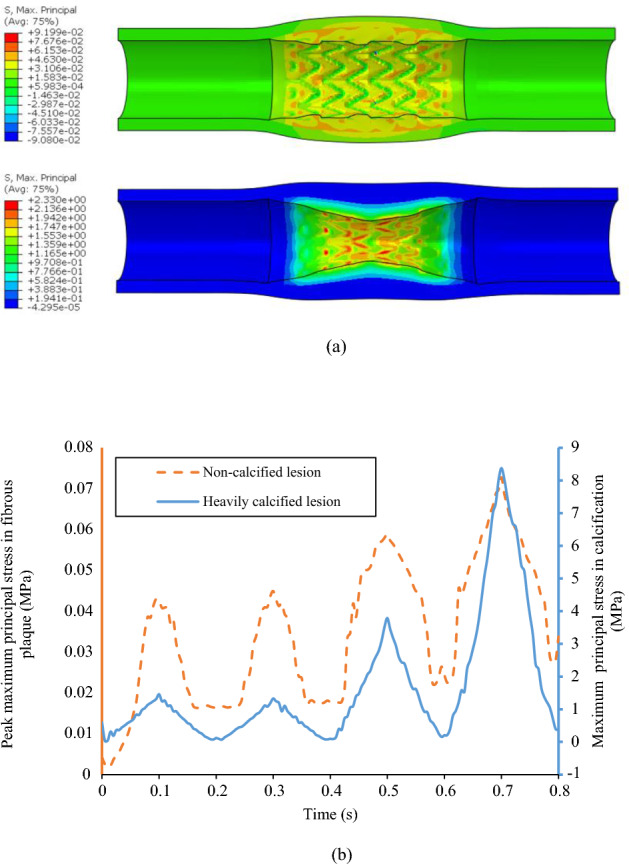
(**a**) Direct stenting-induced maximum principal stress (unit: MPa) maps in non-calcified (top) and heavily calcified lesions (bottom) at the balloon pressure of 10 atm; (**b**) The peak maximum principal stress of plaque for stent expansion in non-calcified lesion case (orange) and heavily calcified lesion case (blue) during stenting and three sequential post-dilations at pressure of 10, 20 and 30 atm.

The distribution of maximum principal stress (MPS) on the lesions at 10 atm was depicted in Fig. 6a. The time history of the peak value of MPS in the non-calcified and heavily calcified lesions during stenting, and the three sequential post-dilations at pressures of 10, 20 and 30 atm, were plotted in Fig. 6b. The peak value of MPS in the non-calcified lesion, occurred in the artery, was 40 kPa during stenting at an inflation pressure of 10 atm, and increased to 70 kPa during the 3^rd^ post-dilation at an inflation pressure of 30 atm. For the heavily calcified lesion, the peak value of MPS, occurred in the calcification, was 1.5 MPa during stenting at an inflation pressure of 10 atm, and increased to 8.4 MPa during the 3^rd^ post-dilation at an inflation pressure of 30 atm. It is worth noting that the MPS time history is consistent with the load sharing analysis (Fig. 5). For the non-calcified lesion, the arterial tissue only stores a small portion of the strain energy and doesn’t increase proportionally along with the increase of the inflation pressure. This indicates a lower risk of vessel rupture. On the contrary, strain energy stored in the calcification increases 14 times as the inflation pressure increases from 10 to 30 atm, indicating a higher probability of calcification fracture.

## Discussion

Suboptimal stenting (stent underexpansion, malapposition etc.) in complex lesions, especially in heavily calcified lesions, has gained increasing attention. Plaque modification and/or post-dilation with a high-pressure balloon has been developed to improve stent expansion and apposition. The cardiologists are under pressure to choose efficient procedures for a target stent expansion with less risk of vessel rupture. In this work, the link between the inflation pressure to the exact force exerted on the inner surface of various lesions was inspected with computer simulations. To our best knowledge, this is the first study to quantify the load transfer of the stent-artery interaction and the load-sharing capacities of the different components during stenting and post-dilation procedures. Results have emphasized the monitoring of the stent expansion during stenting and supported the design of the high-pressure balloon. The stent expansion showed a nearly cylindrical shape in the non-calcified lesion and the balloon undertook most of the increasing inflation pressure load. In contrast, stent expansion showed a dog bone shape in the heavily calcified lesion, suggesting that an increasing inflation pressure can effectively increase the minimal lumen area, increase the stresses in the calcification, and potentially lead to a calcification fracture. The efficacy of the increasing inflation pressure in the heavily calcification lesion is due to the dog bone shape of the stent expansion.

Most previous computational studies of stent-artery interactions focused on the mechanical environment change (i.e., stress and strain) in arterial tissues^18,35^ or stent fracture when exposed to cardiac wall movement following stenting^36^. Our previous works have investigated the influence of calcification attributes on stenting expansion with stylized and patient specific artery models^23,37^, and results have shown that the calcification will reduce the stretch capability of the lesion, further leading to stent underexpansion. In this work, load transfer analyses were conducted for stenting in non-calcified and heavily calcified lesions to quantify the effective load for stent expansion. Only a small portion of the pressure load (1.4 N, 0.8% of the total pressure load) was transferred to the non-calcified lesion. This small portion of the pressure load only induced an effective pressure of 75 mmHg, which is slightly less than the tested blood pressure in a human body, which is around 100 mmHg^38^. Additionally, the balloon pressure for stenting, which is usually around 12 atm (or 9120 mmHg), is much higher than the blood pressure. This drastic difference between the dilation pressure and the exact pressure required for expanding the lesion has previously been ignored in studies on stent-artery interactions. The load transfer and load sharing analysis will build a direct link between the increasing pressure and the effective force to the lesion, which will provide a rational index for optimal stenting. In addition, a radial force of 22.4 N was required to expand the stent, which is similar to the reported studies^39,40^. These agreements validate the feasibility of our model and methods. For the heavily calcified lesion, the concentrated, thick calcification acts as a stiff ring, which induces high restoration forces that prevents the proper expansion of the balloon and causes the observed dog bone shape. For example, the non-calcified lesion can expand to 2.8 mm at 10 atm, while the heavily calcified lesion expands to only 1.48 mm. The balloon which expands in a dog bone shape was not able to undertake as high of a pressure load as the cylindrical shaped balloon. Because of this, a higher-pressure load of 19 N (1027 mmHg) was transferred to the lesion, which is more than 12 times than what is shown in the non-calcified lesion. We can image that as the lesion changes from non-calcified to a mild, medium, and then heavily calcified lesion, the balloon stent expansion will change from a nearly cylindrical shape to a more irregular and/or dog bone shape, during which the pressure load transferred to the lesion will increase from a lower to a higher value. Our previous virtual bench tests also have shown that the resultant contact along the normal of one cut plane increased from 1.5 N to 2.5 N, to 3.5 N as the calcification angle increased from 60º, to 180º, to 270º^41^.

Post-dilation has long been adopted to improve stent expansion by increasing inflation pressure or using a larger balloon. One study has even shown that high pressures up to 40 atm can lead to an improved stent deployment in heavily calcified lesions^7^. Some other retrospective studies, however, have showed that a pressure of 16 atm and a balloon diameter of 0.5 mm larger than the nominal diameter of stent are two critical cap values, and there will be more complications if the inflation pressure exceeds these^3^. In this work, the load sharing analysis for post-dilation with increasing pressure was done to evaluate the efficacy of the procedure. In the non-calcified lesion, the stent expansion has a nearly cylindrical profile (diameter close to manufacturing data) and the balloon undertook most of the pressure load. Therefore, the increasing pressure did not significantly increase the minimal lumen diameter, stress levels, or strain energy within the lesion. For the heavily calcified lesion, the stent expansion has a dog bone shape, and the increasing pressure load can effectively increase the minimal lumen diameter, stress levels and strain energy in the calcification, and the potential of calcification fracture. Based on this observation, increasing pressure can be an effective procedure to improve the stent expansion if a dog bone shape was observed. In addition, a larger balloon may cause the lesion to rupture if the calcification suddenly fractures at a high pressure, which may lead to acute myocardial infarction as reported in clinics^2^. These findings indicate that the monitoring of the stenting process, especially the stenting profile and diameter at the target inflation pressure, may help in making optimal stenting decisions for patients. Our results also support the design and application of the high-pressure balloon. Currently, the burst pressure of most post-dilation balloons is around 20 atm, while it cannot induce a calcification fracture, and the cardiologists shift to a larger balloon, with the risk of vessel rupture. As we noticed that the increasing pressure can effectively exert more force to the lesion, the high-pressure balloon can be a safe way to induce the calcification fracture by increasing the inflation pressure to a higher-pressure limit of the balloon. Computational studies of patient-specific models can aid in estimating the required pressure to induce fracture, as they offer valuable insights into not only the stent deployment but also the more precise assessment of load-sharing characteristics in an in-vivo environment.

In the light of using high-pressure balloons to induce calcification fracture, it is essential to also give due consideration to the mechanical performance of the stent. Higher pressure rates during post-dilation are expected to result in notable stresses and plastic deformations in the vicinity of the strut interconnections, which may lead to stent rupture. According to our simulation analysis, the maximum equivalent plastic strain within the stent rises from 15% during stenting at 10 atm to 30% during post-dilation at 30 atm. Typically, the elongation at break of stents is around 43%, implying the need for careful consideration when selecting the pressure levels^42^. Additional research should be conducted on various stent geometries to assess the practicability of high-pressure applications. Furthermore, this observation may also serve as an opportunity to improve stent design for a given inflation condition, along with the implementation of materials with greater strength and elongation characteristics.

There are some limitations in this work, such as the selection of material model and simulation techniques, as well as simplifications to the overall testing systems and assumptions. The isotropic hyperelastic models were adopted to describe the mechanical behavior of the arterial tissue without considering the anisotropic or viscoelastic behaviors. The plaque was simplified into two materials: fibrosis and calcification. The complexity and eccentricity of the lesion were also simplified as the symmetric model for comparison with the analytical results. The balloon characteristics were also simplified to help better understanding of the load transfer. These simplifications may cause a deviation in the stress analyses but won’t affect the global values such as the resulted force or strain energy, so the conclusions in this work can still be applied for a model with further specificity. The fracture behavior of the calcification was not considered in this work since our focus is the load sharing analysis to provide the fundamental understanding, the fracture behavior will affect the evolution of the restored strain energy. We simulated the balloon with a cylindrical surface, rather than the three-folded balloon based on our benchtop test in which the balloon exhibited a cylindrical shape even at very low pressure (1 atm). Once the inflation pressure exceeded the nominal pressure, the balloon showed a larger resistance. We captured the bilinear behavior of the balloon and verified it with the manufacturer data. Of notice, the findings from this work were derived from idealized model with a single stent type, and the conclusion from this work should be further validated with clinical observations.

## Conclusion

In this work, the diameter-pressure curve and load transfer analysis for stenting in non-calcified and heavily calcified coronary artery were performed. Further load sharing and stress analyses were conducted to investigate the efficacy of increasing the pressure over improving the stenting expansion in heavily calcified coronary artery. The stent expansion showed a nearly cylindrical shape in the non-calcified lesion and the balloon undertook most of the increasing inflation pressure load. In contrast, stent expansion showed a dog bone shape in the heavily calcified lesion, and the increasing inflation pressure can effectively increase the minimal lumen area, stress in the calcification, and potential of calcification fracture. This study suggested that monitoring the stenting process with angiography imaging, especially the precise stent expansion profile at the target inflation pressure, will help optimize the stenting procedure in complex lesions. Our results also support the design and application of the high-pressure balloon for post dilation.

### Supplementary Information

Supplementary Information.

## Data Availability

The datasets used and/or analyzed during the current study are available from the corresponding author on reasonable request.

## References

[1] Seth A, Gupta S, Pratap Singh V, Kumar V (2017). Expert opinion: optimising stent deployment in contemporary practice: the role of intracoronary imaging and non-compliant balloons. Interv. Cardiol.

[2] Zhang Z-J, Marroquin OC, Stone RA, Weissfeld JL, Mulukutla SR, Selzer F, Kip KE (2010). Differential effects of post-dilation after stent deployment in patients presenting with and without acute myocardial infarction. Am. Heart J..

[3] Ortega-Paz L, Capodanno D, Gori T, Nef H, Latib A, Caramanno G, Di Mario C, Naber C, Lesiak M, Capranzano P, Wiebe J, Mehilli J, Araszkiewicz A, Pyxaras S, Mattesini A, Geraci S, Naganuma T, Colombo A, Münzel T, Sabaté M, Tamburino C, Brugaletta S (2017). Predilation, sizing and post-dilation scoring in patients undergoing everolimus-eluting bioresorbable scaffold implantation for prediction of cardiac adverse events: development and internal validation of the PSP score. EuroIntervention.

[4] Brueck M, Scheinert D, Wortmann A, Bremer J, von Korn H, Klinghammer L, Kramer W, Flachskampf FA, Daniel WG, Ludwig J (2002). Direct coronary stenting versus predilatation followed by stent placement. Am. J. Cardiol..

[5] Kocum T, Yurtdas M, Ozcan T, Akcay B, Erol T, Camsari A, Doven O (2008). Direct stenting versus predilatation and stenting technique when using paclitaxel-eluting stents. Int .Heart J..

[6] Kovar LI, Monrad ES, Sherman W, Kunchithapatham S, Ravi KL, Gotsis W, Silverman G, Brown DL (2001). A randomized trial of stenting with or without balloon predilation for the treatment of coronary artery disease. Am. Heart J..

[7] Díaz JF, Gómez-Menchero A, Cardenal R, Sánchez-González C, Sanghvi A (2012). Extremely high-pressure dilation with a new noncompliant balloon. Tex. Heart Inst. J..

[8] Schiavone A, Zhao LG (2015). A study of balloon type, system constraint and artery constitutive model used in finite element simulation of stent deployment. Mech. Adv. Mater. Mod. Proce..

[9] Gervaso F, Capelli C, Petrini L, Lattanzio S, Di Virgilio L, Migliavacca F (2008). On the effects of different strategies in modelling balloon-expandable stenting by means of finite element method. J. Biomech..

[10] De Beule M, Mortier P, Carlier SG, Verhegghe B, Van Impe R, Verdonck P (2008). Realistic finite element-based stent design: the impact of balloon folding. J. Biomech..

[11] Laubrie JD, Mousavi JS, Avril S (2020). A new finite-element shell model for arterial growth and remodeling after stent implantation. Int. J. Numer. Methods Biomed. Eng..

[12] Zheng Q, Dong P, Li Z, Lv Y, An M, Gu L (2020). Braided composite stent for peripheral vascular applications. Nanotechnol. Rev..

[13] Lin S, Dong P, Zhou C, Dallan LAP, Zimin VN, Pereira GTR, Lee J, Gharaibeh Y, Wilson DL, Bezerra HG, Gu L (2020). Degradation modeling of Poly-l-Lactide Acid (PLLA) bioresorbable vascular scaffold within a coronary artery. Nanotechnol. Rev..

[14] Wang R, Zuo H, Yang Y-M, Yang B, Li Q (2017). Finite element simulation and optimization of radial resistive force for shape memory alloy vertebral body stent. J. Intell. Mater. Syst. Struct..

[15] Zheng Q, Dong P, Li Z, Han X, Zhou C, An M, Gu L (2019). Mechanical characterizations of braided composite stents made of helical polyethylene terephthalate strips and NiTi wires. Nanotechnol. Rev..

[16] Mozafari H, Dong P, Zhao S, Bi Y, Han X, Gu L (2018). Migration resistance of esophageal stents: the role of stent design. Compute. Biol. Med..

[17] Chiastra C, Wu W, Dickerhoff B, Aleiou A, Dubini G, Otake H, LaDisa JF (2016). Computational replication of the patient-specific stenting procedure for coronary artery bifurcations: from OCT and CT imaging to structural and hemodynamics analyses. J. Biomech..

[18] Wei L, Chen Q, Li Z (2019). Influences of plaque eccentricity and composition on the stent–plaque–artery interaction during stent implantation. Biomech. Model. Mechanobiol..

[19] Conway C, McGarry JP, Edelman ER, McHugh PE (2017). Numerical simulation of stent angioplasty with predilation: an investigation into lesion constitutive representation and calcification influence. Ann. Biomed. Eng..

[20] Huang X, Yang C, Canton G, Ferguson M, Yuan C, Tang D (2012). Quantifying effect of intraplaque hemorrhage on critical plaque wall stress in human atherosclerotic plaques using three-dimensional fluid-structure interaction models. J. Biomech. Eng..

[21] Gu, L., Zhao, S., Muttyam, A. K., and Hammel, J. M., 2010, The relation between the arterial stress and restenosis rate after coronary stenting. J. Med. Dev. **4**(031005).

[22] Lally C, Dolan F, Prendergast PJ (2005). Cardiovascular stent design and vessel stresses: a finite element analysis. J. Biomech..

[23] Dong, P., Bezerra, H. G., Wilson, D. L., and Gu, L., 2018, Impact of Calcium Quantifications on Stent Expansions. J. Biomech. Eng. **141**(021010). 10.1115/1.4042013 PMC6298533 30453326

[24] Dong, P., Mozafari, H., Prabhu, D., Bezerra, H. G., Wilson, D. L., and Gu, L., 2020, “Optical Coherence Tomography-Based Modeling of Stent Deployment in Heavily Calcified Coronary Lesion,” Journal of Biomechanical Engineering, **142**(051012). 10.1115/1.4045285 PMC7104774 31654052

[25] Barrett HE, Cunnane EM, Hidayat H, O Brien JM, Kavanagh EG, Walsh MT (2017). Calcification volume reduces stretch capability and predisposes plaque to rupture in an in vitro model of carotid artery stenting. Eur. J. Vasc. Endovasc. Surg..

[26] Dong P, Mozafari H, Lee J, Gharaibeh Y, Zimin VN, Dallan LAP, Bezerra HG, Wilson DL, Gu L (2021). Mechanical performances of balloon post-dilation for improving stent expansion in calcified coronary artery: computational and experimental investigations. J. Mech. Behav. Biomed. Mater..

[27] Cejna M (2005). Cutting balloon: review on principles and background of use in peripheral arteries. Cardiovasc. Intervent. Radiol..

[28] Her A-Y, Shin E-S, Bang LH, Nuruddin AA, Tang Q, Hsieh I-C, Hsu J-C, Kiam OT, Qiu C, Qian J, Ahmad WAW, Ali RM (2021). Drug-coated balloon treatment in coronary artery disease: recommendations from an asia-pacific consensus group. Cardiol. J..

[29] BIB® Stent Placement Balloon Catheter | NuMED For Children. Available: https://www.numedforchildren.com/product/bibr-stent-placement-balloon-catheter. [Accessed: 07-Sep-2023].

[30] Martin D, Boyle F (2013). Finite element analysis of balloon-expandable coronary stent deployment: influence of angioplasty balloon configuration: finite element analysis of coronary stent deployment. Int. J. Numer. Meth. Biomed. Engng..

[31] Zhao, S., Gu, L., and Froemming, S. R., 2012. Finite element analysis of the implantation of a self-expanding stent: impact of lesion calcification. J. Med. Dev. **6**(021001).

[32] Liang DK, Yang DZ, Qi M, Wang WQ (2005). Finite element analysis of the implantation of a balloon-expandable stent in a stenosed artery. Int. J. Cardiol..

[33] Gastaldi D, Morlacchi S, Nichetti R, Capelli C, Dubini G, Petrini L, Migliavacca F (2010). Modelling of the provisional side-branch stenting approach for the treatment of atherosclerotic coronary bifurcations: effects of stent positioning. Biomech. Model. Mechanobiol..

[34] Zhao S, Gu L, Froemming SR (2012). On the importance of modeling stent procedure for predicting arterial mechanics. J. Biomech. Eng..

[35] Pericevic I, Lally C, Toner D, Kelly DJ (2009). The influence of plaque composition on underlying arterial wall stress during stent expansion: the case for lesion-specific stents. Med. Eng. Phys..

[36] Morlacchi S, Pennati G, Petrini L, Dubini G, Migliavacca F (2014). Influence of plaque calcifications on coronary stent fracture: a numerical fatigue life analysis including cardiac wall movement. J. Biomech..

[37] Dong, P., Mozafari, H., Prabhu, D., Bezerra, H. G., Wilson, D. L., and Gu, L., 2020, Optical coherence tomography-based modeling of stent deployment in heavily calcified coronary lesion. J. Biomech. Eng. **142**(5). 10.1115/1.4045285 PMC7104774 31654052

[38] Understanding Blood Pressure Readings. www.heart.org. Available: https://www.heart.org/en/health-topics/high-blood-pressure/understanding-blood-pressure-readings. [Accessed: 24-Apr-2022].

[39] Gökgöl C, Diehm N, Nezami FR, Büchler P (2015). Nitinol stent oversizing in patients undergoing popliteal artery revascularization: a finite element study. Ann. Biomed. Eng..

[40] Kumar A, Bhatnagar N (2021). Finite element simulation and testing of cobalt-chromium stent: a parametric study on radial strength, recoil, foreshortening, and dogboning. Comput. Methods Biomech. Biomed. Engin..

[41] Dong, P., Lin, S., Wilson, D. L., Bezerra, H. G., and Gu, L., 2017. Target lesion calcium arc influence the performance of stenting. Am. Soc. Mech. Eng. Dig. Collect. 10.1115/DMD2017-3455 PMC5906068 29683139

[42] Poncin, P., and Proft, J. 2004. Stent Tubing: Understanding the Desired Attributes. In Medical device materials: proceedings of the materials & processes for medical devices conference, pp. 253–259.

