# Supplementary material for: Load-sharing characteristics of stenting and post-dilation in heavily calcified coronary artery
Source: Sci Rep. 2023 Oct 6;13:16878. doi: 10.1038/s41598-023-43160-4 (PMC10558511; doi:10.1038/s41598-023-43160-4)
Supplement: Supplementary file 1 — Supplementary Information. [file 41598_2023_43160_MOESM1_ESM.docx]

Mesh convergence study from [1]

History plot of the Kinetic-to-Internal energy ratio

Heavily Calcified Lesion – Stenting @ 10 atm





Heavily Calcified Lesion – Post-dilation @ 10 atm





Heavily Calcified Lesion – Post-dilation @ 20 atm





Heavily Calcified Lesion – Post-dilation @ 30 atm





Non-calcified Lesion – Stenting @ 10 atm





Non-calcified Lesion – Post-dilation @ 10 atm





Non-calcified Lesion – Post-dilation @ 20 atm





Non-calcified Lesion – Post-dilation @ 30 atm





References

[1] Dong, P., Bezerra, H. G., Wilson, D. L., and Gu, L., 2018, “Impact of Calcium Quantifications on Stent Expansions,” Journal of Biomechanical Engineering, 141(021010).
